# Diversity of Ligninolytic Enzymes and Their Genes in Strains of the Genus *Ganoderma*: Applicable for Biodegradation of Xenobiotic Compounds?

**DOI:** 10.3389/fmicb.2017.00898

**Published:** 2017-05-23

**Authors:** Giselle Torres-Farradá, Ana M. Manzano León, François Rineau, Lucía L. Ledo Alonso, María I. Sánchez-López, Sofie Thijs, Jan Colpaert, Miguel Ramos-Leal, Gilda Guerra, Jaco Vangronsveld

**Affiliations:** ^1^Laboratory of Biotechnology, Department of Microbiology and Virology, Faculty of Biology, University of HavanaHavana, Cuba; ^2^Department of Phytopathology, Research Institute for Tropical Fruit TreesHavana, Cuba; ^3^Environmental Biology, Centre for Environmental Sciences, Hasselt UniversityHasselt, Belgium

**Keywords:** white-rot fungi, *Ganoderma*, laccase, manganese peroxidase, versatile peroxidases, bioremediation, chromophoric compounds, Persistent Organic Compounds

## Abstract

White-rot fungi (WRF) and their ligninolytic enzymes (laccases and peroxidases) are considered promising biotechnological tools to remove lignin related Persistent Organic Pollutants from industrial wastewaters and contaminated ecosystems. A high diversity of the genus *Ganoderma* has been reported in Cuba; in spite of this, the diversity of ligninolytic enzymes and their genes remained unexplored. In this study, 13 native WRF strains were isolated from decayed wood in urban ecosystems in Havana (Cuba). All strains were identified as *Ganoderma* sp. using a multiplex polymerase chain reaction (PCR)-method based on ITS sequences. All *Ganoderma* sp. strains produced laccase enzymes at higher levels than non-specific peroxidases. Native-PAGE of extracellular enzymatic extracts revealed a high diversity of laccase isozymes patterns between the strains, suggesting the presence of different amino acid sequences in the laccase enzymes produced by these *Ganoderma* strains. We determined the diversity of genes encoding laccases and peroxidases using a PCR and cloning approach with basidiomycete-specific primers. Between two and five laccase genes were detected in each strain. In contrast, only one gene encoding manganese peroxidase or versatile peroxidase was detected in each strain. The translated laccases and peroxidases amino acid sequences have not been described before. Extracellular crude enzymatic extracts produced by the *Ganoderma* UH strains, were able to degrade model chromophoric compounds such as anthraquinone and azo dyes. These findings hold promises for the development of a practical application for the treatment of textile industry wastewaters and also for bioremediation of polluted ecosystems by well-adapted native WRF strains.

## Introduction

Environmental pollution with hazardous industrial wastes containing recalcitrant xenobiotics has become a major ecological issue. In contrast to naturally occurring organic compounds that are readily degraded upon their introduction into the environment, some synthetic substances are very resistant to biodegradation by indigenous microorganisms (Asgher et al., 2008). The development of cost effective and at the same time efficient methods for their removal from industrial wastewaters and also from water ecosystems and soils is very important. White-rot fungi (WRF) have the potential to be used as powerful biotechnological tools that can contribute to solve this problem. WRF possess an enzymatic system for lignin degradation, which, due to its broad substrate specificity, has been reported as responsible for the transformation and mineralization of lignin related compounds considered Persistent Organic Pollutants (POPs) such as industrial dyes, chlorophenols, polychlorinated biphenyls, polycyclic aromatic hydrocarbons (PAHs), pesticides and munition wastes (Rodríguez-Couto, 2009; Lanfermann et al., 2015). The main extracellular ligninolytic enzymes are laccases (EC 1.10. 3.2), manganese peroxidase (MnPs, EC 1.11.1.13), lignin peroxidases (LiPs, EC:1.11.1) and versatile peroxidases (VPLs, EC 1.11.1.16) (Janusz et al., 2013; Rivera-Hoyos et al., 2015). Fungal laccases use the redox ability of copper ions to catalyze the oxidation of a broad range of aromatic substrates concomitantly with the reduction of molecular oxygen to water (Giardina et al., 2010). LiPs are able to oxidize high redox-potential aromatic compounds and non-phenolic lignin model dimers while MnPs oxidizes Mn^2+^ to Mn^3+^ (Martínez, 2002). VPLs combine the substrate specificity characteristics of both LiPs and MnPs enzymes (Martínez, 2002; Pérez-Boada et al., 2005). A rapid and practical approach for verifying the presence of this non-specific enzymatic system involved in the degradation of xenobiotic compounds is to determine the capacity of the WRF strains to decolorize model chromophoric compounds (Maganhotto et al., 2005). Model dyes such as Remazol Brilliant Blue R (RBBR) (anthraquinone dye) and Reactive Black 5 (azo dye) have been conveniently used to screen large numbers of fungi with ligninolytic activity and degradative capacity (Levin et al., 2010; Jamal et al., 2011; Zeng et al., 2012). These model dyes have a chemical structure similar to different POPs, therefore these chromophoric compounds are used in biodegradation studies of these xenobiotic compounds.

A majority of the earlier studies focused on the lignin-degrading enzymes of *Phanerochaete chrysosporium* and *Trametes versicolor*. Nowadays there exists a growing interest in finding better lignin-degrading systems to use them in biotechnological applications. The genus *Ganoderma* was extensively investigated because some of its species possess medicinal properties (Park et al., 2012; Kües et al., 2015). However, the potentialities of its ligninolytic machinery have attracted little attention. Nevertheless, some authors reported interesting decolorization properties by some *Ganoderma* sp. strains (Murugesan et al., 2007; Zhuo et al., 2011; Manavalan et al., 2013). Furthermore, it has been described that the majority of the investigated *Ganoderma* strains are able to produce laccase enzymes at higher levels compared with peroxidases (D’Souza et al., 1999; Murugesan et al., 2007; Mendonça et al., 2008; Zhuo et al., 2011). Therefore, the laccase enzymes from different *Ganoderma* strains have been purified and characterized (Ko et al., 2001; Teerapatsakul et al., 2007; Kumar et al., 2015). In addition, there exist a few reports related with the detection of genes coding laccases (D’Souza et al., 1996; Joo et al., 2007; Zhuo et al., 2011; Manzano et al., 2013; You et al., 2013) and peroxidases (D’Souza et al., 1999) from *Ganoderma lucidum*. Zhuo et al. (2011) and You et al. (2013) reported the molecular cloning of a laccase gene from *G. lucidum* and their heterologous expression.

Several authors studied the bioremediation capacity of WRF strains deposited in public collections (Jaouani et al., 2003). However, there have been less investigations attempting to exploit directly local biodiversity (Pointing et al., 2000; Sanchez et al., 2008). However, this approach appears to be potentially productive for identifying new, promising strains for biotechnological applications (Pointing et al., 2003).

In case of the genus *Ganoderma* the studies have been performed using mainly single strains from culture collections and with diverse ecological origins, but the genetic potential and ligninolytic machinery of several well-adapted autochthonous *Ganoderma* strains have not been explored.

A high biodiversity of the genus *Ganoderma* (Minter et al., 2001) and the description of different species such as *G. australe, G. zonatum, G. opacum, G. colossus, G. lucidum, G. coffeatum, G. flaviporum* (Pérez and Camino, 2000; Cabarroi et al., 2008, 2014) and *G. weberianum* (Manzano et al., 2013) have been reported for Cuba. Moreover, Almaguer et al. (2014) described the presence of airborne spores of the *Ganoderma* genus in the atmosphere of Havana as one of the predominant spores of basidiomycetes. In spite of this, the diversity of ligninolytic enzymes and their genes in Cuban native strains of *Ganoderma* genus remain unexplored; only Manzano et al. (2013) described the presence of five new laccase genes and several laccase isozymes present in the strain *Ganoderma weberianum* B-18. Therefore, the study of native strains of *Ganoderma* sp. may lead to an untapped genetic potential for ligninolytic enzymes that could be applied for degradation of POPs. The main objectives of this work are: (1) To analyze the diversity of ligninolytic enzymes and genes of Cuban native strains from the genus *Ganoderma.* (2) To evaluate the contribution of the ligninolytic enzymes to the degradation of model chromophoric compounds.

## Materials and Methods

### Isolation and Identification of WRF Strains Belonging to the Genus *Ganoderma*

The fruiting bodies of basidiomycetes belonging to the genus *Ganoderma* were taken from the base of the trees or decayed wood located in different urban areas (such as parks and main streets) in Havana, Cuba during the years 2013 and 2014. They were identified as *Ganoderma* based on their typical morphology. Pure fungal cultures were isolated from the context of fruiting bodies by using Malt Extract Agar (Merck, Germany) according to the methodology of Guglielmo et al. (2007) and Manzano et al. (2011). Genomic DNA was isolated using Wizard Genomic DNA Purification Kit (Promega, United States). Taxonomic confirmation of the strains was done by means of multiplex polymerase chain reaction (PCR)-based on the amplification of the internal transcriber spacer region of ribosomal DNA (primers ITS 1, ITS 4, **Table 1**) and taxon-specific primers (Gano 2R, **Table 1**) for *Ganoderma* species (Guglielmo et al., 2007). The PCR conditions were as reported Guglielmo et al. (2007). Purified PCR products were sequenced at Macrogen, The Netherlands.

**Table 1:** Sequence of primers used in the present study.

Primers	Nucleotide sequences (5′–3′)	Reference	Specificity
ITS 1	TCCGTAGGTGAACCTGCGG	Guglielmo et al., 2007	ITS-5.8S rRNA gene
ITS 4	TCCTCCGCTTATTGATATGC		
Gano 2R	TATAGAGTTTGTGATAAACGCA		
Cu 1F	CAT(C)TGGCAT(C)GGNTTT(C)TTT(C)CA	Luis et al., 2004	Laccase gene
Cu 2R	G G(A)CTGTGGTACCAGAANGT NCC		
pmnp 1	ACCTTCCACGACGCTATT	Sarkar et al., 1997	Manganese peroxidase gene
pmnp 2	GACATCGGAGCAATCGAT		

*The primers were synthesized at Biolegio (Nijmegen, Netherlands).*

### Determination of Ligninolytic Enzymes and Isozymes Produced by *Ganoderma* Strains

*Ganoderma* strains were cultured in 100 mL Erlenmeyer flasks with 20 mL of SB-U medium (33.9 mL.L^-1^ of sugarcane molasses, 3 g.L^-1^ urea, 1 g.L^-1^ KH_2_PO_4_ and 0.5 g.L^-1^ MgSO_4_.7 H_2_O, pH 5.5, Manzano et al., 2013). The incubation of the strains in SB-medium was performed in agitated conditions at 100 rpm. For determination of the activities of ligninolytic enzymes and isozyme analysis, extracellular crude enzymatic extracts were obtained after removal of the mycelium by filtration (Sartorius 0, 22 μm, United Kingdom) every 24 h.

The laccase activity was determined spectrophotometrically by examining the oxidation of 2 mmol.L^-1^2,6-dimethoxyphenol (DMP) to 2,2′,6,6′-dimethoxydiphenoquinone in 100 mM acetate buffer, pH 5.0 at 468 nm (𝜀468 nm: 49 600 mmol^-1.^L.cm^-1^) (Teerapatsakul et al., 2007). The activity of non-specific peroxidase (NsP) was measured by the oxidation of 0.5 mM *o*-dianisidine in presence of 4 mM H_2_O_2_ in 50 mM of sodium acetate buffer, pH 5 at 445 nm (𝜀445 nm: 47 665 mmol^-1.^L.cm^-1^) (Claiborne and Fridovich, 1979). The enzymatic activities were defined as the amount of enzyme required to produce 1 μmol product.min^-1^ at 30°C and expressed as international units per liter of medium (U.L^-1^).

Isozyme analysis of extracellular crude enzyme extracts was performed by a native polyacrylamide gel electrophoresis (native-PAGE, 15%) (Murugesan et al., 2007). The staining of the gels was performed in 100 mM acetate buffer (pH 5.0) with 10 mM 2,6 dimethoxyphenol (Manzano et al., 2013). Dark orange bands indicated the presence of laccase activity. To detect peroxidase activity the gels were stained with 2, 6 dimethoxyphenol together with H_2_O_2_ (0.1 mmol.L^-1^) and MnSO_4_ (0.1 mmol.L^-1^).

### Diversity of Laccase and Manganese Peroxidase Genes

The analysis of the diversity of laccase and MnPs genes of the *Ganoderma* strains was carried out by means of PCR amplification and sequence analysis. The primers were designed previously taking into account highly conserved regions in DNA sequences of these genes in basidiomycetes (**Table 1**). For the amplifications, 1 μL of the DNA extract was added to a 50 ml reaction mixture containing 5 μL of 10X High Fidelity PCR Buffer, 2 μL of 50 mM MgSO4, 1 μL of dNTP Mix (10 mM each) 1 μL of each primer (10 μM), and 0.2 μL of Platinum^®^ *Taq*DNA Polymerase High Fidelity (5 U.μL^-1^). All the PCR components were obtained from Invitrogen Life Technologies, United States. Genomic DNA of strains *Ganoderma weberianum* B-18 and *Phanerochaete chrysosporium* MUCL 19343 were used as positive controls for the amplification of the laccase and *mnp* genes, respectively. PCRs were run on a Master cycler gradient system (Eppendorf, Hamburg, Germany) with an initial denaturation cycle (2 min at 94°C) followed by 35 cycles with denaturation (30 s at 94°C), annealing for laccase amplification (60 s at 50°C) and for *mnp* amplification (60 s at 53°C) and elongation (2 min at 68°C) and by a final elongation (10 min at 68°C). PCR reactions without DNA were used as negative controls.

The PCR products were separated by electrophoresis in 2% (w/v) agarose gels. PCR products purified from gel were cloned into TOP10 chemically competent *Escherichia coli* after their ligation to a pCR4-Topo Vector according to the manufacturer’s instructions for the TOPO TA Cloning Kit (Invitrogen Life Technologies, United States). The plasmid DNA, containing the PCR product, was extracted from *Escherichia coli* TOP10 by using the GeneJet Plasmid Miniprep Kit (Invitrogen Life Technologies, United States). The purified recombinant plasmids were sequenced using the universal primers (M13for and M13rev) of Macrogen Europe (The Netherlands). Fifteen clones per cloning reaction were sequenced and analyzed (Kellner et al., 2007).

### Evaluation of Decolorization Capacity of *Ganoderma*sp. Strains

The strains were tested for their *in vitro* ability to decolorize model chromophoric compounds such as anthraquinone dyes [Remazol Brilliant Blue R (RBBR), Intraacid Blue 62 (AB-62)] and azo dyes [Reactive Black 5 (RB-5) and Intraacid Navy (INT-R)]. For this purpose we used the extracellular crude enzymatic extracts obtained in SB-medium at the day of the maximun ligninolytic enzyme production. The experiment was carried out in reaction tubes containing 100 mM acetate buffer pH 5, 100 μL of crude enzymatic extracts and 100 mg.L^-1^ of each dye individually. The reaction tubes were incubated in agitation conditions at 100 rpm at 30°C and complete darkness during 12 h (Camarero et al., 2005).

All experiments were performed in triplicate. An abiotic control without enzyme addition was included and a control with the heat inactivated enzyme (autoclaved at 121°C, 15 min) was also used. Decolorization percentages were determined by the difference in absorbance between sample filtrates and abiotic control at the corresponding maximum wavelength for each dye.

### Sequence Analysis

Identical/similar nucleotide sequences were identified by BLAST (Basic Local Alignment Search Tool) search on sequences available online in the databases GenBank, NCBI, EMBL, DDBJ, and PDB. All the sequences were edited and analyzed using CLC Main Work Bench 7.5.1 and Vector NTI 7 software.

To determine the intron positions of the amplified laccase and MnP genes, the obtained sequences were aligned with known cDNA coding laccase or MnP genes from basidiomycetes (e.g., *T. versicolor;* Accession No. L78077). Multiple alignment was performed by means of Clustal W (Thompson et al., 1994) using the following parameters: gap opening penalty = 10, gap extension penalty = 0.2, and Gonnet protein weight matrix. The introns were discarded following the intron splice junctions corresponding to the general eukaryotic rule –5′-GTA/G…..T/CAG-3′ (Kupfer et al., 2004; Luis et al., 2004; Hildén et al., 2005). The protein sequences translated with Vector NTI 7 software were used for phylogenetic analysis.

The phylogenetic analyses were performed by the Maximum Likelihood Analysis and Bayesian Analysis with software Mega 7.0 (Kumar et al., 2016) and BEAST (Bayesian Evolutionary Analysis Sampling Trees (BEAST) v 1.8.4, Drummond et al., 2012) respectively. The Tracer software v1.6.0 (Rambaut et al., 2014) was used to analyze graphically and quantitatively the empirical distributions of continuous parameters obtained after the analysis with BEAST. The FigTree application v 1.4.3 (Rambaut, 2014) was used for displaying the molecular phylogenies.

### Statistical Analysis

Results are presented as the average of five replicates. Normality and variance homoscedasticity were investigated prior to carrying out the statistical analyses by means of the Kolmogorov-Smirnov test and the Bartlett test, respectively. Where data met these criteria, an analysis of variance of simple classification (ANOVA) and a parametric Tukey’s test were used. If these preliminary criteria were not fulfilled, the Kruskal–Wallis and SNK tests were used. All data were processed with the statistical package Statistic 7.0.

## Results

### Isolation of Indigenous White-Rot Fungi Belonging to the Genus *Ganoderma*

Thirteen native *Ganoderma* fruiting bodies were collected from decayed wood in Havana (Cuba) based on morphological characteristics of this genus such as the presence or absence of laccate or shiny appearance of the upper surface of the fruiting bodies (Zakaria et al., 2009).

**FIGURE 1:**
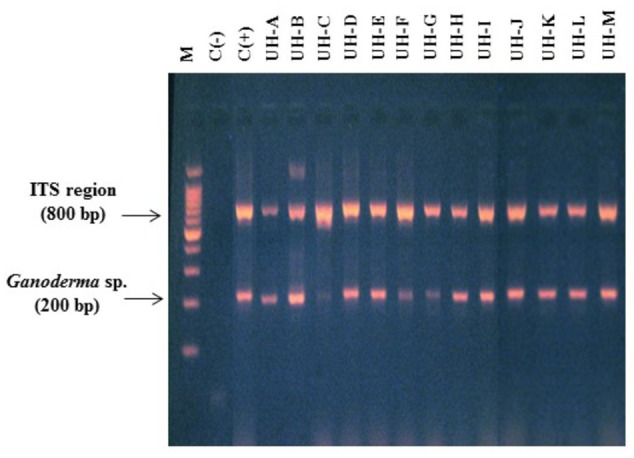
**Multiplex polymerase chain reaction (PCR) (primers ITS 1, ITS 4, Gano 2R) for the identification of WRF strains.** Lane M. Molecular marker 100 bp Promega. Lanes 1 and 2. Negative and positive controls. Lanes 3–15: strains UH-A until UH-M.

The fungal isolates that were collected were named consecutively as UH-A until UH-M and were deposited in the Collection of Microbial Cultures of Faculty of Biology, University of Havana (CCMFB) with consecutive numbers from CCMFB-H714 until CCMFB-H726. The multiplex PCR showed that all the isolated strains were belonging to the genus *Ganoderma* (**Figure 1**).

The sequencing of the ITS regions and the analysis of DNA sequences through the BLAST-N program with nucleotide sequences previously published in NCBI GenBank showed that all the ITS sequences obtained from the WRF strains were 99% identical to ITS sequences of different strains of *Ganoderma* (accession numbers in Gen Bank: JQ514104.1, GQ249880.1, JQ514105.1, HM192933.1, AF255133.1, KF963258.1, JN637827.1, KC884264.1). **Table 2** shows the accession numbers of ITS sequences from *Ganoderma* strains.

**Table 2:** Accession numbers of DNA sequences obtained in this work.

*Ganoderma* sp. strains	Accession numbers EMBL			
	ITS	Laccase	Manganese peroxidase	Versatile peroxidase
*Ganoderma weberianum* **B-18** CCMFB-H601	JN637827	*lac* I HE585213	–	LT726744
		*lac* IIHE585214		
		*lac* III HE585215		
		*lac* IV HE585216		
		*lac* V HE585217		
**UH-A** (CCMFB-H714)	LT726719	*lac* I LN999878	LT726718	–
		*lac* II LN999879		
		*lac* III LN999880		
		*lac* IV LN999881		
**UH-B** (CCMFB-H715)	LT726720	*lac* I LN999882	–	LT726737
		*lac* II LN999883		
		*lac* III LN999884		
		*lac* IV LN999885		
**UH-C** (CCMFB-H716)	LT726721	*lac* I LN999886	LT726732	–
		*lac* II LN999887		
		*lac* III LN999888		
		*lac* IV LN999889		
**UH-D** (CCMFB-H717)	LT726722	*lac* I LN999890	–	LT726736
		*lac* II LN999891		
**UH-E** (CCMFB-H718)	LT726723	*lac* I LN999892	–	LT726738
		*lac* IILN999893		
**UH-F** (CCMFB-H719)	LT726724	*lac* ILN999894	–	LT726743
		*lac* IILN999895		
		*lac* III LN999896		
**UH-G** (CCMFB-H720)	LT726725	*lac* ILN999897	–	LT726739
		*lac* IILN999898		
		*lac* IIILN999899		
**UH-H** (CCMFB-H721)	LT726726	*lac* ILN999900	LT726733	–
		*lac* II LN999901		
**UH-I** (CCMFB-H722)	LT726727	*lac* ILN999902	LT726734	–
		*lac* II LN999903		
		*lac* III LN999904		
**UH-J** (CCMFB-H723)	LT726728	*lac* I LT796160	LT726735	
		*lac* II LT796161		
		*lac* III LT796157		
**UH-K** (CCMFB-H724)	LT726729	*lac* I LN999905	–	LT726742
		*lac* II LN999906		
		*lac* III LN999907		
UH-L (CCMFB-H725)	LT726730	*lac* ILN999908	–	LT726740
UH-M (CCMFB-H726)	LT726731	*lac* I LT796162		LT726741
		*lac* II LT796158		
		*lac* III LT 796163		
		*lac* IV LT 796159		

### Determination of Ligninolytic Enzymes and Diversity of Laccase Isozymes

In order to determine the ligninolytic enzymes produced by the *Ganoderma* strains and also to define the day of the maximum production of these enzymes, a time course analysis of laccase and non-specific peroxidases (NsP) production was performed in SB-U medium with sugarcane molasses as carbon source and ligninolytic enzyme inducer (Manzano et al., 2013).

**FIGURE 2:**
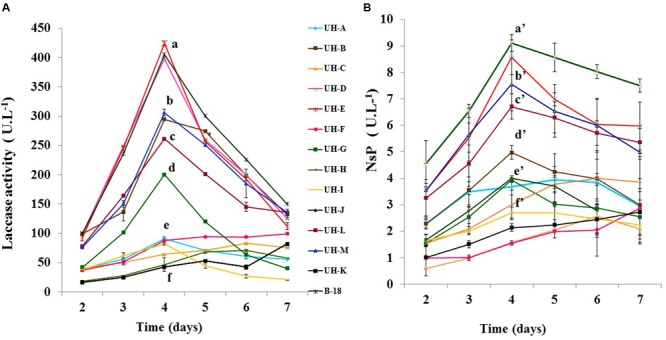
**Time course analysis of (A)** laccase **(B)** Non-specific peroxidases (NsP) production by *Ganoderma* sp. strains in SB-U medium. The cultures were kept at 30°C during 7 days. The results show the means ± SD of five independent cultures. Different letters indicate significant differences (*p* < 0.05) according to Tukey HSD test.

All strains were able to produce NsP and laccase enzymes (**Figure 2**). The activities of both enzymes were identified from the 2nd day of incubation. For most of the *Ganoderma* strains the highest productions of both ligninolytic enzymes were reached at the 4th day of incubation. The strains UH-E, B-18, UH-D, UH-M, UH-B, and UH-L showed the highest values for both enzymes. In all strains the levels of laccase activities (42.2–424.3 U.L^-1^) were one order of magnitude higher than those of the NsP (1.5–9.1 U.L^-1^).

**FIGURE 3:**
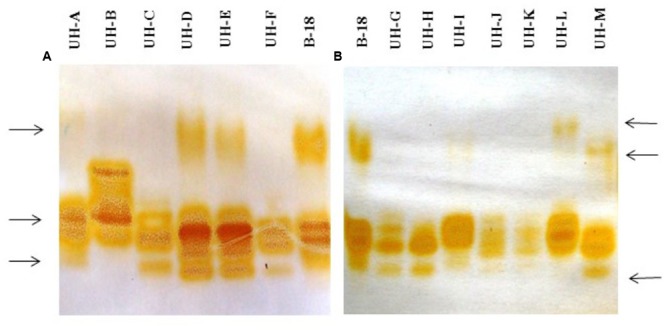
**Native polyacrylamide gel electrophoresis analysis of extracellular crude enzymatic extracts of *Ganoderma* strains. (A)** *Ganoderma s*trains UH-A until UH-F. **(B)** *Ganoderma strains* UH-G until UH-M. Staining with 2,6 dimetoxyphenol.

The analysis of extracellular crude enzymatic extracts of *Ganoderma* strains by native-PAGE and staining with an enzyme specific substrate allowed the detection of several laccase isozymes, with diverse isozymatic patterns that differ in numbers, mobility and intensity of the electrophoretic bands (**Figure 3**).

Not all the strains produced the same laccase isozymes. The main differences were the following: there was a band with less electrophoretic mobility in the strains B-18, UH-A, UH-D, UH-E, UH-L, and UH-M; however, there were some differences in the electrophoretic mobility of this isoform between the strains UH-M and UH-L. There was a high-intensity band that it was only present in the strain UH-B. Finally, the strains B-18, UH-D, UH-E, UH-F, UH-G, UH-H, and UH-M produced the same band with a lower electrophoretic mobility.

Although NsP activity was detected in extracellular enzyme extracts, it was not possible to detect other orange zones when gels were stained with the 2,6 DMP assayed together with H_2_O_2_ and MnSO_4_, which are favorable conditions for the catalytic action of ligninolytic peroxidases.

### Diversity of Genes Encoding Ligninolytic Enzymes of *Ganoderma* Strains

#### Amplification of Laccase Gene

**FIGURE 4:**
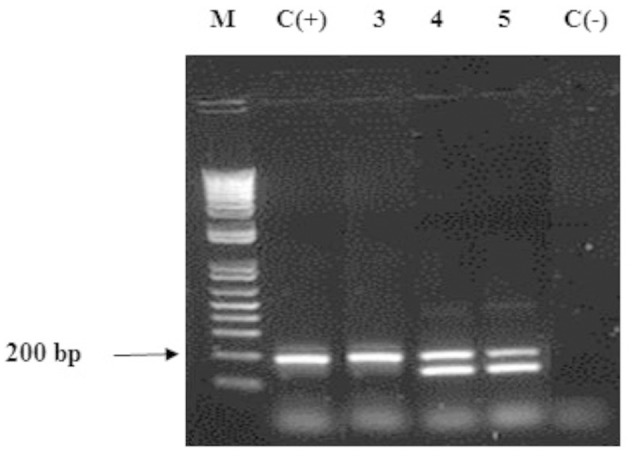
**Agarose gel electrophoresis (2% w/v) of fragments of laccase genes amplified by PCR on *Ganoderma* strains.** Lane M: DNA ladder mix (1kb, Invitrogen), lane 2: positive control *Ganoderma weberianum* B-18. Lanes 3–5: *Ganoderma* UH. strains (illustration of the 13 strains). Lane 6: negative control.

Taking into account that all the *Ganoderma* strains produced several laccase isozymes and to obtain a better knowledge of the laccase system of *Ganoderma* strains, we investigated if the laccase isozymes detected in these strains are coded by one or more laccase genes and also examined the novelty of their sequences. PCR products obtained with the primers Cu1F/Cu2R showed DNA fragments of sizes ranging from 144 to 240 bp (**Figure 4**), which was consistent with the expected amplicon size (Luis et al., 2004). In some cases we obtained more than one band in the agarose gel electrophoresis.

The analysis of DNA sequences through the BLAST-N program with nucleotide sequences published in NCBI GenBank allowed to distinguish different laccase genes in each *Ganoderma* UH strain (EMBL Nucleotide Sequence Database LN999879 - LN999908, **Table 2**). The laccase genes detected were 80–87% identical to laccase genes of *Ganoderma weberianum* (*lac* 1: HE585213.1, *lac* 2: HE585214.1, *lac* 4:HE585216.1*), Ganoderma lucidum* (KC507947.1, DQ914870.1, JN654464.1), *Trametes sp*. (AY846842.1), *Flammulina velutipes* (AY485826.1), *Lenzites gibbosa* (JF817353.1) and uncultured basidiomycetes (AJ420341.1, EU882652.1).

**FIGURE 5:**
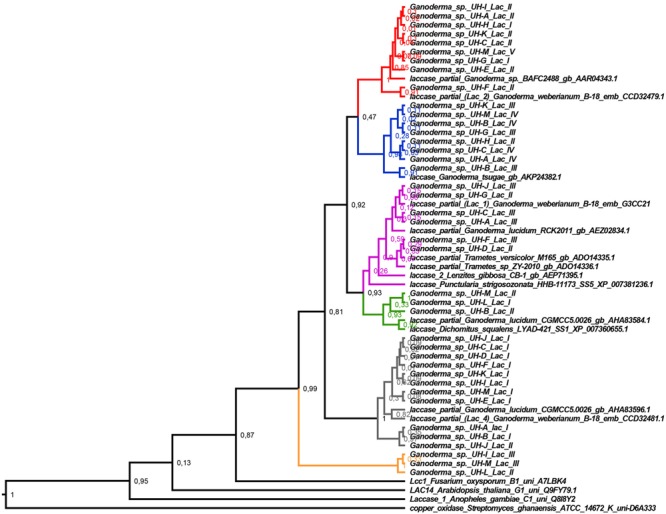
**Bayesian tree of deduced amino acid sequences of laccase gene fragments from the *Ganoderma* strains and the closely related sequences retrieved from GenBank after the Blast P analysis.** Posterior probability values are shown in each node. The red, blue, violet, green, gray, and orange branches indicate the different specific clades that are formed. The Bayesian tree was constructed with BEAST v 1.8.4 with the following parameters: substitution model: WAG model with Gamma distribution, tree prior: Yule process, Markov chain Monte Carlo: length of chain 20 000 000. The rest of parameters were set as default options.

To analyze the diversity of the laccase genes detected, a phylogenetic tree (**Figure 5**) was constructed with the translated amino acid sequences of laccase gene fragments from *Ganoderma* strains and closely related laccase protein sequences retrieved from GenBank after the Blast P analysis. A representation of sequences belonging to the different laccase families (Sirim et al., 2011) was included in the analysis (Family B: ascomycete *Fusarium oxysporum* B1 uni A7LBK4, Family G: plant *Arabidopsis thaliana* G1 uni Q9FY79.1, Family C: insect *Anopheles gambiae* C1 uni Q8I8Y2 and Family K: the copper oxidase *Streptomyces ghanaensis* ATCC 14672 K. Phylogenetic trees obtained by Bayesian analysis and Maximum Likelihood correspond in terms of the general tree topology; here, we only present the Bayesian tree with the posterior probability in each node.

Interestingly, the laccase genes detected in the *Ganoderma* strains did not group in a common clade. Six principal clades were formed where the laccase genes of different strains were grouped together with other laccase protein sequences belonging to the Family A of laccase (Basidiomycete Laccase) according to The Laccase Engineering Database^1^ (Sirim et al., 2011). The major clades that were formed are supported with high posterior probability values after the Bayesian analysis. This fact confirms the differences between these laccases.

A relatively high diversity of laccase genes was found among the *Ganoderma* strains. All strains have at least two highly distinctive laccase gene sequences. This supports the detection of several laccase isozymes in all *Ganoderma* strains. The laccase partial sequences grouped in each clade have identical amino acid sequences, with the exception of the sequences UH-K Lac II, UH-F Lac II, UH-B Lac II. This fact was confirmed by multiple alignment of these sequences and pairwise distances analysis.

**FIGURE 6:**
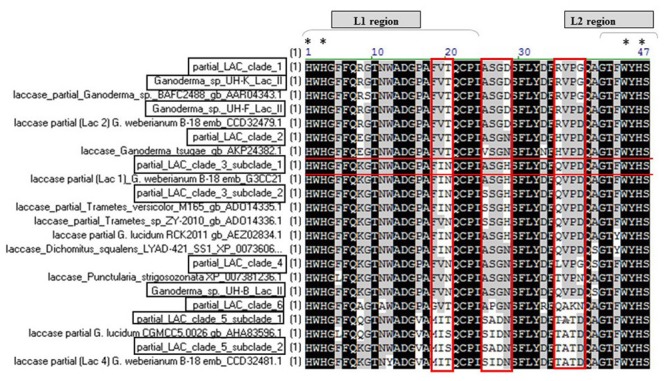
**Extracted fragment from the multiple alignment the deduced amino acid sequences of laccase fragments from *Ganoderma* strains with the laccases presented in the phylogenetic tree.** The position residues conserved in all sequences are highlighted in black. The positions conserved in 80 and 60% of the sequences, respectively, are highlighted in dark and light gray. The L1 region and a section of L2 region present in the studied fragments as well as the histidine residues involved in copper-binding (T2 and T3-sites ligands marked with asterisks) of the active site are indicated. LAC clade 1 (UH-I lac II, UH-A lac II, UH-H lac I, UH-K lac II, UH-C lac II, UH-M lac V, UH-G lac I, UH-E lac II), LAC clade 2 (UH-M lac IV, UH-B lac IV, UH-G lac III, UH-H lac II, UH-C lac IV, UH-A lac IV, UH-B lac III), LAC clade 3 subclade 1 (UH-C lac III, A lac III, G lac II), LAC clade 3 subclade 2 (UH-F lac III, UH-D Lac II), LAC clade 4 (UH-M lac II, UH-L lac I), LAC clade 5 subclade 1 (UH-J lac I, UH-C lac I, UH-D lac I, UH-F lac I, UH-K lac I, UH-I lac I, UH-M lac I, UH-E lac I), LAC clade 5 subclade 2 (UH-A lac I, UH-B lac I, UH-J lac II) LAC clade 6 (UH-I lac III, UH-M lac III, UH-L lac II).

**Figure 6** illustrates the multiple alignment of the laccase deduced amino acid sequences of *Ganoderma* strains with 11 of the laccases presented in the phylogenetic tree. The alignment showed that all the laccase proteins detected in UH strains contained the conserved histidine residues involved in copper-binding regions I and II (T2 and T3-sites ligands) of the active site (Giardina et al., 2010) as well as the L1 region with the patterns [H-W-H-G-x(9)–D-G-x(5)–Q-C-P-I] and a section of the region L2 (GTFWYHS).

In the L1 and L2 regions, considered as signature regions of laccase enzymes (**Figure 6**) the copper ligand residues HWHG and WYHS of the L1 and L2, respectively, are highly conserved in all the sequences, however, not all the amino acids of the L1 region are preserved in all laccase proteins from the *Ganoderma* strains. This is especially evident if we consider the residues FVT, ASGD and RVPG (marked in red) which are not conserved in all the laccase sequences of *Ganoderma* UH strains.

With the exception of partial laccases that are grouped in clade 3 subclade 2 (sequences UH-F lac III, UH-D Lac II, marked in red in **Figure 6**), which is 100% identical to a partial lac 2 sequence of *Ganoderma weberianum* B-18, the rest of the partial laccase protein sequences from the studied *Ganoderma* strains showed differences in their amino acid sequences with respect to laccases previously described in GenBank. The most divergent protein and with more novelty in its sequence are the partial laccase proteins grouped in clade 6 (sequences UH-I lac III, UH-M lac III, UH-L lac II).

#### Amplification of Gene Encoding Manganese Peroxidase

**FIGURE 7:**
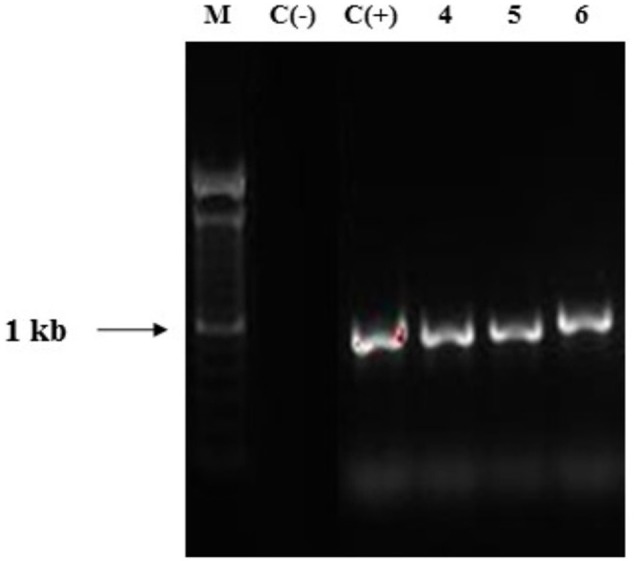
**Agarose gel electrophoresis of PCR products obtained with primers pmnp1/ pmnp2.** Lane 1: DNA ladder mix (1kb, Invitrogen), Lane 2: negative control. Lane 3: positive control *Phanerochaete chrysosporium* MUCL 19343 Lanes 4–6 *Ganoderma* strains (representative for the 13 strains).

**FIGURE 8:**
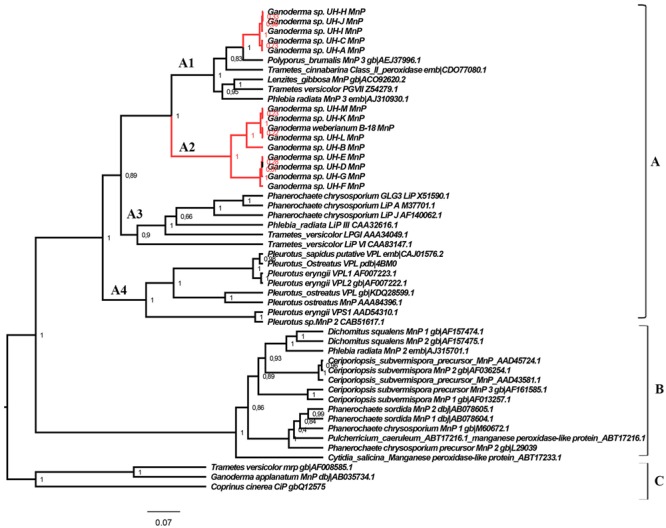
**Phylogeny of the class II fungal secretory heme peroxidases.** The main phylogenetic subgroups A, B, and C and translated peroxidases sequences of *Ganoderma* strains are illustrated. Posterior probability values are shown in each node. The Bayesian tree was constructed with BEAST v 1.8.4 with the following parameters: substitution model: LG model with Gamma distribution, tree prior: Yule process, Markov chain Monte Carlo: length of chain 20 000 000. The rest of parameters were set as default options.

Polymerase chain reaction using primers pmnp1 and pmnp2 amplified the expected 1.0 kb fragment from the genomic DNA of all *Ganoderma* UH strains (**Figure 7**). The analysis using the BLAST-N program and multiple alignment of the cloned sequences revealed that in contrast to laccases, only one gene encoding peroxidases was present in all *Ganoderma* sp. strains (**Table 2**). In order to analyze the phylogenetic relationship with previously studied fungal peroxidases, a Bayesian tree (**Figure 8**) was constructed with the translated amino acid sequences of genes encoding peroxidases detected in the UH strains, the sequences retrieved from GenBank after the Blast P analysis and sequences that belong to class II peroxidases.

The translated peroxidases enzymes detected in the *Ganoderma* strains were clustered in group A of class II peroxidases (Hildén et al., 2005), but in two different clades: clade A1 and clade A2. In group A we can also find sequences of LiPs (clade A3), VPL (clade A4) and the short-type hybrid MnPs variants (clades A1, A4) more related to LiPs and VPLs than to the classical long MnPs (group B). The sequences of group C are structurally related, but are functionally different from the ligninolytic enzymes forming the majority of class II fungal peroxidases.

**FIGURE 9:**
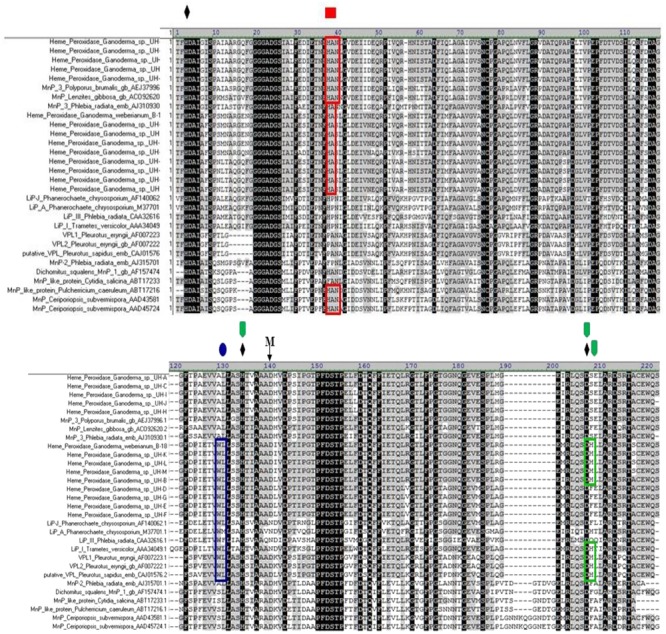
**Multiple amino-acid sequence alignment of the deduced amino acid sequence of MnP from *Ganoderma* strains in comparison to other deduced fungal manganese peroxidases.** Functional residues described in the text are marked with symbols:distal and proximal histidines, **M-** manganese binding residues, 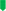 and aminoacids marked in green; 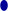 and aminoacids marked in blue ; 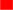 and aminoacids marked in red are residues proposed to be active in the LRET I, LRET II and LRET III pathway respectively

Interestingly, the sequences of peroxidases detected in *Ganoderma* UH strains were related to short-type hybrid MnPs variants, but were also associated to LiPs sequences of *Phanerochaete chrysosporium, T. versicolor*, and *Phlebia radiata* (**Figure 8**). To determine the similarities that peroxidases identified in this work share with the LiPs and VPLs studied previously we performed a multiple alignment analysis (**Figure 9**) with sequences of these peroxidases grouped in different clades of the phylogenetic tree obtained. In the alignment we searched for the presence or absence of the active site residues for manganese binding characteristic of manganese dependent enzymes (MnPs) and the critical residues for the three different long-range electron transfer (LRET) pathways proposed for the substrate oxidation that LiPs and VPLs can perform (Pérez-Boada et al., 2005; Morgenstern et al., 2008).

In all the predicted amino acid sequences of *Ganoderma* UH strains, the catalytically indispensable amino acids within the heme environment were found. The two histidines (heme peroxidase proximal H132 and distal H3) and the aspartate D 206 were identified in the alignment as ♦. These residues are conserved in all class II peroxidases.

In all partial sequences of peroxidases from UH strains it was possible to identify one residue involved in Mn-binding, the D 138 (M in the alignment). The other two residues involved in Mn-binding were not amplified with the primers used. The amino acid (D138) is conserved in all the translated amino acid sequence obtained from UH strains as well in the sequence VPL1 and VPL2 of *Pleurotus eryngii*. This residue is not present in that position in the sequences of LiPs analyzed.

Residues required for the LRET I pathway are present only in the peroxidases sequences of the strains UH-L, UH-K, UH-M, B-18, and UH-B as well in all the sequences of LiPs of *T. versicolor* (AA34049.1) and in VPLs of *Pleurotus eryngii* (AF007223.1, AF007222.1) and *Pleurotus ostreatus* (CAJ015762) described previously. The critical tryptophan residue required for the LRET II pathway is present in all sequences of *Ganoderma* strains grouped in clade A2 as well as in all sequences of the LiPs and VPLs analyzed. However, it is missing from the sequences of clade A1 and from sequences of MnPs grouped in clade A1 and in group B. The residues hypothetically involved in the LRET III pathway are present in all the sequences analyzed in the alignment, however, there are some differences between the sequences from clade A1 and A3.

Taking into account that the peroxidases grouped in clade A2 from strains UH-L, UH-M, UH-K, UH-B, UH-D, UH-E, UH-G, UH-F, B-18 contain the typical manganese binding residues characteristics of MnPs and also the residues involved in the LRET II described experimentally in LiPs and VLPs, we can consider these sequences as putative versatile peroxidases (VLPs) at functional level because these enzymes combine the catalytic properties of LiPs and MnPs due to the presence of Mn-binding residues and oxidation sites such as LRET described previously.

The peroxidases sequences of *Ganoderma* sp. strains grouped in clade A1: strains UH-H, UH-I, UH-J, UH-C, UH-A have in their sequence the LRET III, however, this oxidation pathway has not been demonstrated experimentally in VPLs or LiPs. Therefore, the peroxidases of these strains can be classified as Manganese Peroxidases enzymes taking into account that in their sequence the Mn binding site is present.

### Decolorization Capacity of *Ganoderma* sp. Strains

**FIGURE 10:**
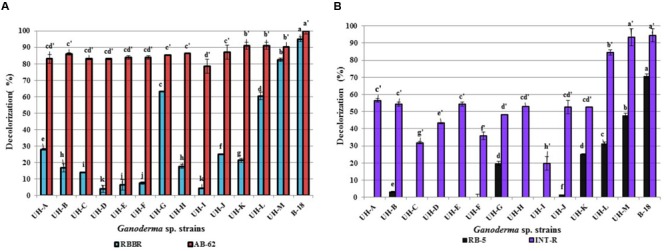
**Decolorization percentages of 100 mg.L^-1^of (A)** anthraquinone dyes **(B)** azo dyes, reached by extracellular crude enzymatic extracts produced by the *Ganoderma* strains. The results show the means ± SD of five independent cultures. Different letters indicate significant differences (*p* < 0.05) according to Tukey HSD test.

The extracellular crude enzymatic extracts, obtained at the day of maximum ligninolytic enzyme production by the *Ganoderma* strains, showed able to decolorize the model anthraquinone and azo dyes that were examined (**Figure 10**).

All *Ganoderma* strains proved capable to degrade differentially the four examined model dyes. The anthraquinone dye AB-62 was degraded to a large extent by all strains (78–100%) followed by the azo dye INT-R (19–94%). The anthraquinone dye RBBR was also degraded by all the strains, but with lower decolorization percentages. Only four strains (UH-G, UH-L, UH-M, and B-18) accomplished decolorization percentages higher than 60% for this dye. The azo dye RB-5 showed to be the most recalcitrant to degradation; only the strains UH-M and B-18 obtained 47 and 70% of decolorization, respectively, the other five strains (UH-B, UH-G, UH-J, UH-K, and UH-L) presented low decolorization values (between 1 and 31%).

## Discussion

In the present study, we isolated 13 basidiomycetes belonging to the genus *Ganoderma* from urban ecosystems in Havana, Cuba. The native strains exhibited differences between the activities of ligninolytic enzymes, the isozyme profiles and the genes encoding for laccase, MnPs and VPLs enzymes. Extracellular crude enzymatic extracts produced by the *Ganoderma* strains showed able to degrade the model chromophoric compounds such as anthraquinone and azo dyes, making them candidates for further exploration in function of treatment of wastewater from the textile industry and/or for bioremediation of matrices polluted with POPs.

The strains were isolated in Havana because this city is one of the most contaminated urban areas in Cuba due to the presence of industrial activities, for example the Refinery of Crude Oil. Moreover, the air of the city is polluted with the exhaust of old cars and busses. For all these reasons, the WRF that grow in such kind of ecosystems are exposed to a whole range of chemical compounds (Lorenzo and Lázaro, 2003). These fungi should be adapted also to conditions of high temperatures and low humidity in comparison with natural ecosystems (Sanchez et al., 2008).

After identification of the WRF strains as *Ganoderma* sp., their degradative capacity was characterized taking into account their capability to produce high activity levels of ligninolytic enzymes, the diversity of the genes coding for these ligninolytic enzymes and the decolorization efficiency of these enzymes against model chromophoric compounds.

Some WRF produce all three ligninolytic enzymes while others produce only one or two of them (Wesenberg et al., 2003). The SB-U medium with sugarcane molasses as carbon source and inducer of ligninolytic enzymes (Manzano et al., 2013) allowed to grow the *Ganoderma* strains and also the production of ligninolytic enzymes. Sugarcane molasses is a byproduct of sugar production, it contains numerous compounds like sugars, amino acids, proteins, vitamins, minerals allowing fungal growth but also contain phenolic acids and aromatic compounds derived from lignin that stimulate production of ligninolytic enzymes (Otero et al., 2000). Due to the composition of the SB-U medium and the inducing effects of their components, the maximum ligninolytic production was reached after 4 days of incubation by all *Ganoderma* strains (**Figure 2**). From the point of view of an eventual industrial application, the use of the SB-U medium offers obvious economic advantages since it allows to reach in a shorter time the maximum ligninolytic production by WRF strains and its components such as urea, KH_2_PO_4_, MgSO_4_.7 H_2_O have low cost and the sugarcane molasses are available in all sugar producing countries.

All *Ganoderma* strains produced laccase enzymes at higher levels than non-specific peroxidases (**Figure 2**); this suggests that the *Ganoderma* strains secreted laccases as dominant ligninolytic enzymes in the conditions used for cultivation. Our results agree with earlier findings reporting high levels of laccase production in other *Ganoderma* strains (Teerapatsakul et al., 2007; Deswal et al., 2012; Fang et al., 2015). The laccase activities produced by *Ganoderma* strains B-18, UH-E, UH-D, UH-M, UH-B, UH-L, and UH-G in SB-U medium were similar and even higher in comparison to laccase activities produced in different culture conditions by other WRF basidiomycetes, including *Ganoderma* strains. For example Novotny et al. (2001), Kokol et al. (2007), and Šnajdr and Baldrian (2007) reported laccase values between 0.31 and 29.4 U.L^-1^ for promising WRF strains such as *Irpex lacteus, Ischnoderma resinosum, T. versicolor*, and *Pleurotus ostreatus*. Laccase activities of 27, 80, and 120 U.L^-1^ were produced by *Ganoderma* sp. (Maganhotto et al., 2005), *Ganoderma australe* (Mendonça et al., 2008), and *Ganoderma* sp. En3 (Zhuo et al., 2011). All these WRF strains were able to decolorize synthetic dyes.

Often, more than one isoform of ligninolytic enzymes is expressed by different WRF depending on the fungal species and the environmental conditions (Majeau et al., 2010; Yuan et al., 2016). The *Ganoderma* strains produced different types and numbers of laccase isozymes in the SB-U medium (**Figure 3**). The strains UH-B, UH-D, UH-E, UH-L, and UH-M presented higher numbers of electrophoretic bands in comparison with the other strains (UH-A, UH-C, UH-F, UH-G, UH-H, UH-J, and UH-K). Moreover, they showed a similar isoenzyme pattern to the *Ganoderma weberianum* B-18 strain (used as a positive control in this experiment). The presence of these laccase isoforms in the cathodic region of the electrophoresis gel could contribute to the higher levels of laccase enzyme detected in the strains UH-B, UH-D, UH-E, UH-L, and UH-M. Moreover, the presence of these laccase isoforms with lower electrophoretic mobility could reflect an adaptive value for the degradation of a wide range of complex substrates. The occurrence of a higher number of laccase isozymes in these *Ganoderma* strains indeed could be advantageous for the degradation of a broad spectrum of xenobiotic pollutants. Several authors (Teerapatsakul et al., 2007; Kumar et al., 2015) demonstrated that single *Ganoderma* strains produced different extracellular isoforms of laccases using diverse culture conditions and ligninolytic inducers. However, our study is the first concerning diversity of laccase isozymes in several strains of the genus *Ganoderma* isolated from urban ecosystems.

The diversity of laccase isozymes in our *Ganoderma* UH strains was associated with the presence of various laccase genes. Between two and five laccase genes were amplified from the genome of each strain (**Figure 5**) and at least one translated sequence of a laccase enzyme with novelty in its sequence was detected in each strain, especially in the region where the residues responsible to maintain a local three-dimensional fold characterizing the active site are located. For example, the residues of L1 region marked in red in the alignment analysis (**Figure 6**) are related with the formation of the one of water channels of laccase enzymes. These channels are very important for the catalytic activity of laccases because they allow the access of the molecular oxygen (final electron acceptor) to the active site of the enzyme and also allow the expel of the water molecules produced (Giardina et al., 2010). The fact that we found differences in these important residues may indicate that the laccase isozymes detected in the *Ganoderma* strains might have novel biochemical properties.

The existence of multiple genes encoding peroxidases has also been described in different ligninolytic fungi (Hofrichter et al., 2010; Järvinen et al., 2012; Janusz et al., 2013). However, in the *Ganoderma* strains only one peroxidase encoding gene was detected in each strain. The phylogenetic analysis (**Figure 8**) and multiple alignment examination of translated proteins (**Figure 9**) revealed differences between the peroxidase encoding genes of the different strains. The peroxidases detected in strains UH-H, UH-I, UH-J, UH-C, and UH-A were classified as manganese peroxidases, however, the translated peroxidases of the strains UH-L, UH-M, UH-K, UH-B, UH-D, UH-E, UH-G, UH-F, and B-18 were classified as putative versatile peroxidase taking into account the presence of residues involved in Mn-binding and residues related with the long-range electron transfer (LRET) pathways described for LiPs and VPLs (Pérez-Boada et al., 2005; Morgenstern et al., 2008).

The molecular structure of ligninolytic peroxidases comprises a heme cofactor situated at an internal cavity (the “heme pocket”) connected to the protein surface through two small access channels. The narrow heme channel prevents the direct contact between the large lignin polymer and the heme. This implies the participation of low molecular mass compounds as peroxidase redox mediators, like the fungal metabolite 3,4-dimethoxybenzyl alcohol (veratryl alcohol). However, even such small compounds appear to be too big to approach the heme through the above-mentioned channel(s). Therefore, LRET pathways from the exterior of the enzyme to the heme cofactor represent a reasonable alternative to explain oxidation of redox mediators, aromatic substrates, and even polymeric lignin performed by LiPs and VPLs enzymes (Pérez-Boada et al., 2005; Morgenstern et al., 2008). Different studies applying crystallographic models (Pérez-Boada et al., 2005; Sundaramoorthy et al., 2005) and site-directed mutagenesis (Gelpke et al., 2002; Pérez-Boada et al., 2005) demonstrated experimentally the occurrence of the LRET I and II pathways in VPLs from *Pleurotus eryngii* (Pérez-Boada et al., 2005; Ruiz-Dueñas et al., 2009) and in LiPs from *Phanerochaete chrysosporium* (Ruiz-Dueñas et al., 2001). However, the LRET III is absent from the crystal structure of the VPLs of *Pleurotus eryngii* and has not been confirmed experimentally in LiPs (Morgenstern et al., 2008). None of the above pathways exists in the crystal structure of MnP of *P. chrysosporium*. However, the peroxidase sequences of the present study that were classified as manganese peroxidases belonging to the strains UH-H, UH-I, UH-J, UH-C, and UH-A, the residues hypothetically involved in LRET III are present. Our results correspond with the analysis performed by Morgenstern et al. (2008) who found after Bayesian analysis and multiple alignment analysis of several MnPs retrieved from GenBank that some “classical” MnPs peroxidases (belonging to group B in the phylogeny of our work) contain the residues hypothetically involved in LRET III, for example the MnP from *Ceriporiopsis subvermispora* (AAD43581, AAD45724), *Cytidia salicina* (ABT17233), and *Pulcherricium caeruleum* (ABT17233). Morgenstern et al. (2008) postulated that if all of the predicted LRET pathways are functional (which still has to be demonstrated experimentally for the vast majority of sequences), then true MnPs may be less widespread than previously thought.

The deduced peroxidase proteins of strains UH-L, UH-M, UH-K, UH-B, UH-D, UH-E, UH-G, UH-F, and B-18 were classified as putative versatile peroxidases due to the presence of the residues related to Mn-binding and also the critical tryptophan residue related to LRET II pathway. These results are similar to the findings of Morgenstern et al. (2008) with a sequence of *Ganoderma applanatum* (AB035734.1) annotated as MnP in GenBank and two MnPs sequences of *T. versicolor* (AAB63460, CAG39281) that possess both manganese binding residues and residues for LRET II and, in fact, might be VPL. These results suggest that VPL might be much more widespread than presently assumed.

The sequences of the peroxidase encoding genes detected in this study have not been described before. These enzymes could be candidates for functional studies in order to determine more precisely the residues involved in the complex catalytic sites of these peroxidases. To the best of our knowledge, the diversity of genes coding ligninolytic enzymes such as laccases, MnPs, and VPLs of different indigenous strains of the genus *Ganoderma* isolated from Cuban urban ecosystems has not been characterized before.

The presence of different genes encoding laccase isozymes in *Ganoderma* strains may have an adaptive value for these WRF that grow on complex substrates such as hard wood, and also in changing environments (Luis et al., 2004; Shleev et al., 2007; Majeau et al., 2010) such as the urban ecosystems from where they were isolated. The presence of these laccase isozymes together with the peroxidases described in this study could provide to these strains multiple advantages to survive in their natural habitats, but might also be valuable for the degradation of a variety of recalcitrant environmental pollutants such as POPs.

Indeed, all crude enzymatic extracts produced by the *Ganoderma* strains could degrade, with diverse percentages, the model chromophoric compounds with chemical structure similar to different POPs compounds after 12 h of incubation (**Figure 10**). However, the dyes RBBR and RB-5 were the most recalcitrant to degradation. The observed differences in decolorization abilities of these WRF suggest differences in the catalytic properties of the ligninolytic enzymes produced. In addition, the differences in chemical structure between these dyes can affect their biodegradability (Levin et al., 2010). Generally, azo dyes are more recalcitrant to biodegradation due to the presence of sulfonate groups and azo bonds (Rodríguez-Couto, 2007). However, we found that the azo dye INT-R was easily degraded by *Ganoderma* strains in comparison to the anthraquinone dye RBBR. This is an interesting result, since it has been reported that azo dyes were recalcitrant to the decolorization or could only be decolorized to a limited extent (Nyanhongo et al., 2002; Levin et al., 2010).

Taking into account that *Ganoderma* strains B-18, UH-L, and UH-M reached the highest percentages of decolorization against the four assayed dyes (between 90 and 100% for AB-62; between 60 and 96% for RBBR, 85–95% for INT-R and 32–70% for RB-5), these strains can be considered as promising candidates for development of an efficient biological system for the treatment of textile industry wastewaters. The values of decolorization achieved by these strains are comparable or superior to those reported by other authors using promising WRF strains for dye degradation. *Phanerochaete chrysosporium, Bjerkandera* sp. and *Irpex lacteus*, for instance, were reported to decolorize the dye RBBR with values of 83, 65, and 90%, respectively (Máximo and Costa-Ferreira, 2004). Correspondingly, Zeng et al. (2011) reported that the extracellular crude extract produced by *Trametes trogii* decolorized the dye RBBR with 85%, but only in presence of the redox mediator 1-hydroxybenzotriazole.

Several studies investigating the decolorization of the diazo-dye RB-5demonstrated that this dye is extremely recalcitrant to degradation due to its complex chemical structure and high redox potential (Camarero et al., 2005; Murugesan et al., 2007). Laccases produced by *Pycnoporus cinnabarinus* and *Trametes villosa* showed able to degrade RB-5 with 70 and 80% of decolorization only in presence of the redox mediators syringaldehyde and acetosyringone (Camarero et al., 2005). In contrast, the extracellular crude enzymatic extracts produced by *Ganoderma* strains B-18, UH-M, and UH-L in our study realized efficient decolorization without the addition of a redox mediator. This is very important taking into account that the laccase-mediator system has yet to be applied on the industrial scale, the cost of mediators and the lack of studies that guarantee the absence of toxic effects of these compounds or their derivatives (Malarczyk et al., 2009).

The fact that all *Ganoderma* strains isolated in this work could degrade the model dyes under investigation might indicate that the ligninolytic machinery of these strains also possesses the capacity to degrade other xenobiotic compounds with similar chemical structures. These findings could open new perspectives for the development of practical applications for degradation of xenobiotic compounds by well-adapted native WRF strains.

## Conclusion

Thirteen native WRF strains were isolated from trees in urban ecosystems in Havana (Cuba). Multiplex PCR with taxa-specific primers and sequence analysis provided strong evidence for classification of all strains as *Ganoderma* sp. All strains produced laccase as the main ligninolytic enzyme in the crude enzymatic extract although non-specific peroxidase activity was also detected. A PCR and cloning approach using specific primers for basidiomycetes allowed determining the diversity of genes encoding laccase and peroxidase enzymes. The *Ganoderma* UH strains possess at least one laccase gene with novelty in their sequence, especially in the region where the amino acid residues are involved in maintaining a local three-dimensional fold characterizing the active site of the enzyme. The diversity of the laccase genes detected in the *Ganoderma* strains indicates the occurrence of several laccase isozymes. In contrast, in each strain, only one gene encoding peroxidases such as MnPs or VPLs was detected. Most of the translated amino acid sequences obtained in the present study have not been described before. All *Ganoderma* UH strains possess the potential to degrade POPs. The present findings contribute to a better understanding of the properties of a WRF genus such as *Ganoderma* which have not been studied in details by other authors. These findings could also constitute a good incentive for better protection of fungal biodiversity.

## Author Contributions

Design of the work: GT-F, AMML, MR-L, MIS-L, JC, GG, and JV. Conducting experiments: GT-F, AMML, LLLA, FR, and ST. Interpretation of data: GT-F, AMML, LLLA, FR, MR-L, MIS-L, GG, JC, ST, and JV. Drafting the work: GT-F, AMML, FR, JC, and JV. Final approval: GT-F, AMML, MR-L, GG, and JV.

## Conflict of Interest Statement

The authors declare that the research was conducted in the absence of any commercial or financial relationships that could be construed as a potential conflict of interest.

## Footnotes

1 http://www.lcced.uni-stuttgart.de
